# Improvement in the ability to have sex in patients with Peyronie's disease treated with Collagenase *Clostridium histolyticum*

**DOI:** 10.1002/bco2.185

**Published:** 2022-10-12

**Authors:** Jeannette P. Geelhoed, Olivier Wegelin, Ellen Tromp, Bert‐Jan de Boer, Igle‐Jan de Jong, Jack J. H. Beck

**Affiliations:** ^1^ St. Antonius Ziekenhuis Nieuwegein The Netherlands; ^2^ University Medical Center Utrecht Utrecht The Netherlands; ^3^ GGD regio Utrecht Utrecht The Netherlands; ^4^ Mannen Gezondheid en Zorg de Boer Bilthoven The Netherlands; ^5^ University Medical Center Groningen, Department of Urology University of Groningen Groningen The Netherlands

**Keywords:** clostridium collagenase histolyticum, intralesional injections, Peyronie's disease, sexual dysfunction, vacuum pump therapy, Xiapex

## Abstract

**Objective:**

To describe the results of intralesional Collagenase *Clostridium histolyticum* (CCH) treatment in patients with Peyronie's disease (PD) in real‐world setting. PD is characterized by curvature of the erect penis caused by fibrotic tissue in the tunica albuginea.

**Patients and methods:**

Patients with stable PD and curvature of 30° to 90° were prospectively enrolled. CCH injections were initially given using a scheme of four cycles of two injections within 48–72 h every 6 weeks. Later using a modified scheme of three injections every 4 weeks, combined with a vacuum erection device (VED) twice daily. All patients were requested to take pictures of the erect penis prior to and following treatment, from above and laterally. Curvature was measured by three independent researchers based on the provided pictures using a goniometer. Furthermore, patients filled in the Peyronie Disease Questionnaire‐NL (PDQ‐NL) and Patient Reported Outcome Measurement (PROM).

The primary outcome was reduction in curvature and the ability to have penetrating sex again. Secondary outcomes include pain scores during injections, changes in PDQ‐NL, PROM and complications of CCH treatment.

**Results:**

Sixty‐three patients were included, mean age was 56.0 years (range 39–70) and mean reduction in curvature 20.6° (SD 10.2, range 5–49); 74.5% of the patients were able to have penetrating sex again following treatment, compared with 41.2% prior to treatment. According to the PROM questions, sexual improvement was seen in 66.7% of patients. The satisfaction rate was 6.8 (SD 1.8). All patients save two recommend treatment.

**Conclusions:**

Intralesional treatment with CCH in men with PD leads to a mean curvature improvement of 20.6°. Following treatment, 74.5% of men were able to have sexual intercourse and 54.9% of the couples were satisfied with their sex life. No major complications occurred in the patients treated with CCH. CCH is not available in Europe anymore despite good results.

## 1 INTRODUCTION

Peyronie's disease (PD) is characterized by a fibrotic tissue disorder of the tunica albuginea in the corpora cavernosa of the penis. The fibrotic tissue in PD contains collagen type I and III. It causes deformity of the erect penis and commonly a palpable nodule. The fibrotic tissue causes painful erections in the early phase of the disease in 20% to 70% of men.^1^ Deformity of the penis can cause hindrance during sexual intercourse and can lead to sexual disability when penetration is not possible anymore. PD also causes psychological distress.^2^, ^3^, ^4^ Often, erectile dysfunction is experienced as a side effect.^5^ PD typically affects men between 40 and 70 years with a prevalence of 1% to 13% amongst all ages.^6^, ^7^, ^8^, ^9^

Collagenase *Clostridium histolyticum* (CCH) contains a mixture of class I and II collagenases in a defined ratio. Its mechanism of action is cleavage of the triple helix of the collagen fibres type I and III.^10^, ^11^ These collagen fibres are present in the tunica albuginea in the penis. The IMPRESS I and II trials have shown its safety and efficacy in patients with PD.^10^ CCH is injected into the dorsal PD nodule of the penis during the stable phase of PD. The original IMPRESS trial treatment scheme contained four cycles of two injections within 48–72 h every 6 weeks during 6 months together with modelling the penis.^10^ The modified scheme introduced by Abdel Raheem et al. contained three injections 4 weeks apart during 3 months together with the use of a vacuum erection device (VED) twice daily, stretching the penis five times for 2 min.^12^, ^13^, ^14^, ^15^, ^16^, ^17^ The treatment of PD with CCH in the acute phase showed good results, even with a single injection.^18^, ^19^, ^20^

Treatment with CCH in patients with stable PD has been introduced in the Netherlands in 2017. The St. Antonius Hospital is the only clinic in the Netherlands offering this conservative treatment option for patients with PD, being the expert centre for PD in the Netherlands till CCH was withdrawn from the European market. CCH treatment is covered by the health insurance for this hospital, based on negotiation. The hospital received a recognition for male sexual diseases by the Dutch foundation for top clinical hospitals in 2021. The researchers were forced to stop the treatment of CCH in patients with PD due to withdrawal from the European market by the manufacturer since March 2020. This present study reports the results in curvature reduction and patient reported outcomes of CCH treatment in patients with stable PD in a real‐world setting.

## 2 PATIENTS AND METHODS

### 2.1 Study design

A prospective observational analysis of all PD patients treated with CCH was performed using real‐world data. All CCH treatment was performed in a single, large nonacademic teaching hospital, regarded as a national PD expert centre.

Patients referred to the outpatient clinic with stable PD and a palpable nodule were eligible for CCH treatment. Inclusion criteria for CCH treatment included a palpable nodule dorsal or lateral of the penis, a stable curvature of 30° to 90° during at least 6 months and difficulty with sexual intercourse. Additional ultrasound examination was not routinely performed because in daily practice, PD is diagnosed by physical examination and history taking. All patients provided signed informed consent for anonymous use of their medical history, treatment results and adverse events. From January 2018 till July 2018, patients were offered the treatment scheme in accordance to the IMPRESS I and II study protocols for injection therapy with CCH.^10^ After publication by Abdel Raheem et al., the modified schedule was introduced July 2018 into the clinic. This schedule contains less visits to the outpatient clinic and less injections and is therefore more patient friendly and more efficient.^12^, ^13^

The original (IMPRESS) scheme contained four cycles of two injections within 48–72 h every 6 weeks during 6 months. The patient had to stretch and bend the penis at home during treatment according to the instructions of the urologist.^10^ The modified scheme contained three injections 4 weeks apart during 3 months. Patients were instructed to use the VED twice daily, mechanically stretching the penis five times for 2 min. Furthermore, patients were instructed to perform home modelling of the penis through gently stretching by hand for 60 s after urinating.^12^

The first 12 patients were treated according to the original IMPRESS scheme. The next 51 patients were treated according the modified scheme as described. All patients were instructed to avoid sexual intercourse for 2 weeks in both schemes. All patients were requested to take pictures of the erect penis prior to and following treatment, from two angles (from above and laterally). The pictures were self‐taken by the patients and were used to measure the difference in curvature following treatment. The curvature degree was measured independently by three researchers (JG, JB and BJB) using a goniometer suitable for measuring curvature to perceive interobserver reliability. In one patient, the curvature prior to treatment was measured with an Alprostadil‐induced erection, whereas the post‐treatment measurement was based on self‐made photo at the outpatient clinic.

Patients were requested to complete the Peyronie's Disease Questionnaire‐NL (PDQ‐NL) before and after treatment. Six weeks following the last injection during an appointment at the outpatient clinic, patients were asked orally standardized questions regarding Patient Reported Outcome Measurement (PROM); see Table 1. Answers were noted in the electronic patient file. The PROM is designed to assess improvement of sexual functioning in PD patients and their partners. It is not validated before use.

**TABLE 1:** PROM questions

1	Was sexual intercourse possible *before* the treatment? (primary outcome) Yes/No
2	Is sexual intercourse possible *after* the treatment? (primary outcome) Yes/No
3	Is the sex better *after* treatment? Yes/No
4	Are you satisfied with the treatment? Yes/No
5	Would you recommend the treatment to another person? Yes/No
6	Is your sexual partner content about the result of the treatment? Yes/No
7	What grade would you give the result of the treatment? 0–10

If injections were too painful, patients were offered a 10cc lidocaine 2% injection prior to CCH injection. If concurrent erectile dysfunction was present at baseline, patients were offered PDE5‐Inhibitor treatment. Penile traction therapy was not offered during the treatment with CCH.

### 2.2 Treatment and measurements

All injections were administered by the same urologist (JB). Patients received injections of 0.4 ml with 0.9 mg CCH into the nodule in the penis. Primary outcome measurements were the measured improvement in curvature and improved capability of performing penetrating intercourse after treatment based on the PROM question. Secondary outcomes were reported complications, a PROM including overall satisfaction rate on a 0 to 10 scale and PDQ‐NL. The PDQ was designed to quantitatively assess the symptoms and psychosexual consequences of PD by providing three subscale domain scores, including PD psychological and physical symptoms (six items), penile pain (three items) and PD symptom bother (four scored items and two yes/no questions).^21^ The PDQ‐NL is not validated in Dutch yet. Pain score was measured after each injection on a numeric rating score (NRS) from 0 to 10, being the most commonly used scale to measure pain. Complications were reported according to Clavien–Dindo score.^22^, ^23^

### 2.3 Statistical method

Data are presented as means (±SD), medians (range or IQR) or count (%), as appropriate.

The interrater reliability of the curvature degree measurements by three researchers was tested by the intraclass correlation coefficient (model: mixed and type: consistency).

The paired *T* test was used to compare mean curvature and differences in means of change in curvature degree and percentage, mean score of PDQ questionnaires' domains (PDQ‐PS, PDQ‐PP and PDQ‐BD) before and after treatment. The possibility of penetrating intercourse before and after treatment was analysed using a McNemar test. We did not perform a power analysis on the number required patients since this is an observational study. Furthermore, the number of patients could not be increased, since CCH is not available anymore for daily practice.

The NRS pain score, overall satisfaction and comparison of the PROM questions between groups were analysed using the Mann–Whitney *U* test.^21^ Missing values of NRS were imputed using multiple imputation. Missing values were replaced with values computed by adding up the mean change of that pain score for all other cases by the score before the missing value.

Demographic characteristics were compared between groups with original and modified treatment schemes using the chi‐square test, Fisher's exact test and independent samples *T* test or the Mann–Whitney *U* test in cases of non‐parametric data. A *p* value of <0.05 was considered statistically significant. The IBM Statistical Package for the Social Sciences (IBM Corp, Released 2015, IBM SPSS Statistics for Windows, version 27.0; IBM Corp.) was used for all statistical analyses.

## 3 RESULTS

**FIGURE 1:**
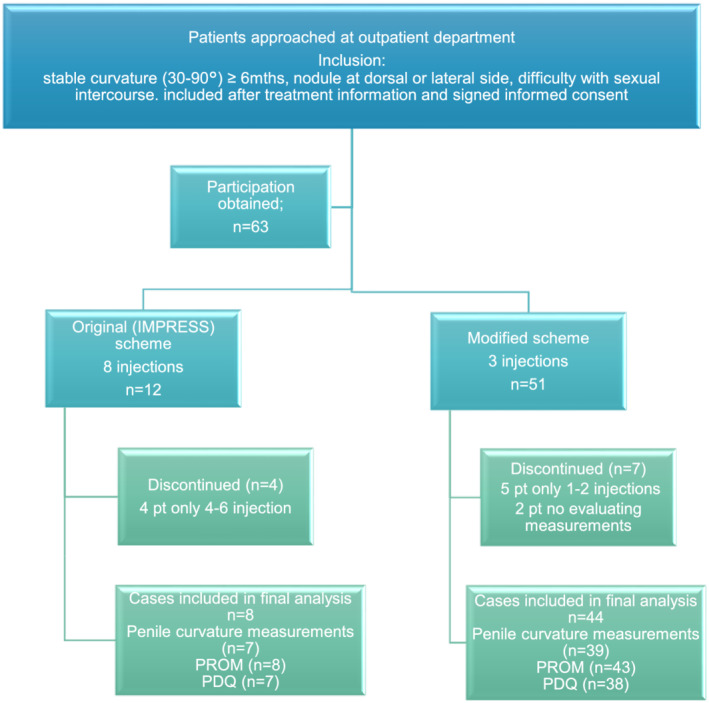
Flowchart of inclusions; pt = patients

A total of 63 patients were treated with CCH in the period between January 2018 and August 2020. Of the 63 patients, 12 were treated according to the original scheme (19%) and 51 using the modified scheme (81%). Eleven patients did not complete treatment according to protocol: four patients in the original treatment scheme (4/12 = 33%) and seven in the modified treatment scheme (7/51 = 14%) (see Figure 1). The main reason to discontinue treatment was that injections were too painful, a more severe coexisting illness, lost to follow‐up and not willing to provide a photograph.

Baseline characteristics are shown in Table 2. The mean patient age was 56.0 years (SD 6.3; range 39–70). The mean penile curvature at baseline was 63.4° (SD 14.4; range 35° to 95°), and 18 patients (40%) were not able to achieve penetrative intercourse based on PDQ Question 1.

**TABLE 2:** Baseline characteristics of the study population

Baseline characteristics	Total (*n* = 52)	Original scheme (*n* = 8)	Modified scheme (*n* = 44)
Age (years), mean (SD; *n*)	56.0 (6.3; 52)	52.3 (8.0; 8)	56.7 (5.8; 44)
Penile curvature (°), mean (SD; *n*)	63.4 (14.4; 46)	67.1 (18.8; 7)	62.7 (13.7; 39)
Severity of penile curvature of ≥60°, *n* (%), *n* = 46	27 (58.7)	5 (71.4)	22 (56.4)
Penetrative intercourse is possible *n* (%), *n* = 45 based on PDQ, Q1	27 (60.0)	5 (71.4)	22 (57.9)

### 3.1 Primary outcomes

A total of 46 treated patients had taken pictures of the erect penis prior to and following treatment. The mean curvature (*n* = 46) prior to treatment was 63.4° (SD 14.4) and after the treatment 45.2° (SD 15.1), mean improvement of 18.2°, *p* < 0.01 following treatment. Of these, 41 patients (89%) showed improvement in the degree of curvature by a mean of 20.6° (SD 10.2, range 5–49). Following treatment, intercourse was possible in 74.5% (*N* = 38) based on the PROM questions. Prior to treatment, only 41.2% (*N* = 21) of the patients were able to have intercourse; see Table 3. All patients whom could have sexual intercourse prior to treatment could have intercourse following treatment.

**TABLE 3:** Results of PROM questionnaire taken after treatment, *n* = 51

PROM questions	*N* (%)
Was sexual intercourse possible *before* the treatment? (primary outcome) Yes	21 (41.2)
Is sexual intercourse possible *after* the treatment? (primary outcome) Yes	38 (74.5)^a^
Is the sex better *after* treatment? Yes	34 (66.7)^a^
Are you satisfied with the treatment? Yes	28 (54.9)
Would you recommend the treatment to another person? Yes	49 (96.1)^b^
Is your sexual partner content about the result of the treatment? Yes	27 (54.0)^c^

^a^ For 3 ptn, this question is not applicable.

^b^ For 1 ptn, this question is not applicable.

^c^ For 6 ptn, this question is not applicable.

The intraclass correlation coefficient of curvature evaluation by three independent researchers was 0.79 (95% CI 0.69–0.87) prior to treatment and 0.78 (95% IC 0.67–0.86) following treatment.

**FIGURE 2:**
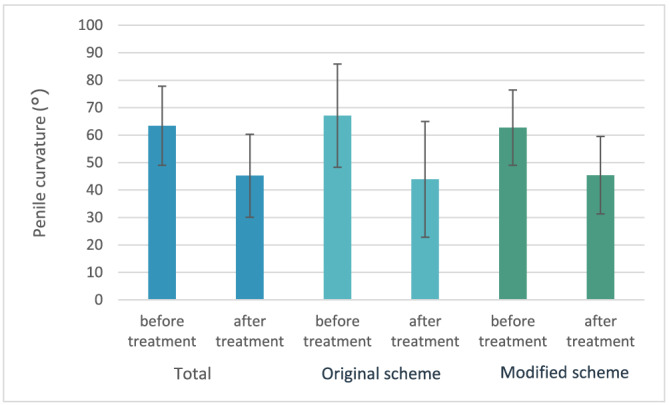
Mean penile curvature before and after treatment, total *n* = 46; original scheme *n* = 7; modified scheme *n* = 38

Additionally the difference between the two treatment schemes was analysed. The improvement in curvature following treatment was significant for both treatment groups separately (original scheme from 67.1° [SD 18.8] to 43.9° [SD 21.1], *p* = 0.01 and modified scheme from 62.7° [SD 13.7] to 45.4° [SD 14.1], *p* < 0.001) (see Figure 2). The mean improvement in curvature was 23.2° (SD 16.4) in the original treatment group and 17.3° (SD 10.7) in the modified treatment group. Fourteen per cent of the patients in the original group and 10% in the modified group did not show an improvement. The curvature improvement was not significantly different between the two groups for degrees (*p* = 0.09) and for percentages (*p* = 0.17).

The percentage of patients with severe penile curvature (>60°) decreased from 59% to 13%. Of the treated patients that provided pictures, three patients (6.5%) showed worsening of the curvature and another two patients (4.3%) showed no change in curvature.

### 3.2 Secondary outcomes

#### 3.2.1 PROM questionnaire

The results of PROM questionnaire are presented in Table 3. Patients who reported better sex after treatment, treatment satisfaction and satisfaction of sexual partner had a higher improvement of the curvature. Reported better sex after treatment showed an improvement of the curvature of 20.0°; SD 11.1 versus 12.3°; SD 13.6, *p* = 0.067. Reported higher treatment satisfaction showed an improvement of curvature of 21.2°; SD 12.2 versus 13.8°; SD 10.3, *p* = 0.041. Reported higher satisfaction of sexual partner showed improvement of curvature: 22.9°; SD 12.2 versus 12.4°; SD 8.2, *p* = 0.006; 96.1% of patients would recommend the CCH treatment. There was no significant difference between the original and modified scheme regarding all the PROM questions.

#### 3.2.2 PDQ‐NL

**FIGURE 3:**
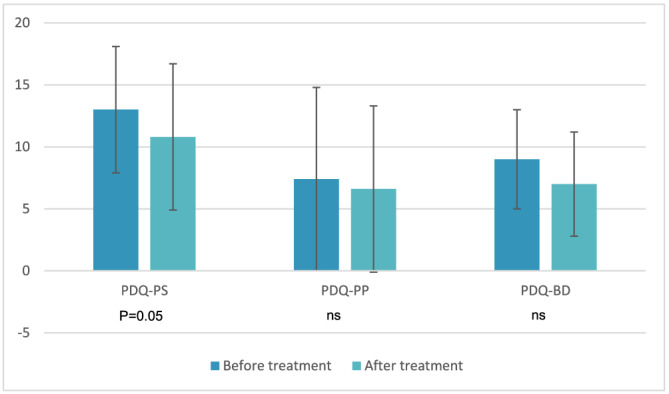
Peyronie's Disease Questionnaire (PDQ) at baseline and after treatment, *n* = 25. PS, physical and psychological symptoms domain; PP, penile pain domain; BD, bother domain

PDQ‐NL was completed by 25 patients prior to and following treatment. There was a non‐significant improvement in all PDQ domains. The first domain, containing the severity of the problems with vaginal intercourse (PDQ‐PS), changed from 13.0 (SD 5.1) to 10.8 (SD 5.9), *p* = 0.053. The second domain experienced pain in flaccid and erect condition for the last 24 h and pain during intercourse (PDQ‐PP), changed from 7.4 (SD 7.4) to 6.6 (SD 6.7), *p* = 0.572. The third domain, containing questions about problems with the erection and vaginal intercourse (PDQ‐BD), altered from 9.0 (SD 4.0) to 7.0 (SD 4.2), (see Figure 3).

Before treatment, 27 patients (60.0%) were able to have penetrative intercourse within the last 3 months before treatment (PDQ Question 1). Following treatment, intercourse was possible in 35 patients (83.3%), five patient (100%) of the original scheme and 30 patients of the modified group (81%). Sexual improvement was seen in ten patients (23.8%) while one patient (2.3%) reported a worsening. The change in ability to have penetrative intercourse was significant (*p* = 0.008). In men who could have intercourse following treatment, a significant reduction in percentage of curvature is seen (21.0°; SD 11.8) in comparison with men who could not have intercourse following treatment (8.3°; SD 8.6), *p* = 0.028.

The overall satisfaction rate of the treatment amongst patients was 6.8 (SD 1.8; range 1–10) and the NRS 4.3 (SD 1.8). The treatment satisfaction was not correlated to NRS (*R* = 0.05, *p* = 0.75) or to a reduction of curvature (*R* = 0.28, *p* = 0.08). Seven patients received on request a pain block of 10 ml lidocaine 2% into the penis before the injections.

Patients who could have sexual intercourse following treatment showed a significant better satisfaction rate (7.1; SD 1.6) than patients who could not have intercourse following treatment (4.2; SD 2.2), *p* = 0.001.

In the group treated with the modified scheme, five patients were motivated to receive an extra cycle of three injections. These patients had a mean improvement in curvature of 40% following the first cycle of treatment. Three of them provided a picture after the first and second series of injections. They showed no further reduction of the curvature.

### 3.3 Complications

One patient developed a skin infection at the injection site (dermatitis) which required antibiotic treatment (Clavien–Dindo 2). Small or larger hematoma in the penis or discolouring of the penile skin due to the VED (Clavien–Dindo 1) was seen in 39 patients. No grade 3–5 complications occurred.

## 4 DISCUSSION

This is the first paper describing the treatment effects of CCH for PD in a real‐world setting amongst Dutch men. The observed outcomes were reduction in curvature and functional outcomes of CCH treatment using PROMs. The mean reduction in curvature is 18.2° compared with 17° in the original studies of Gelbard et al. of eight injections in total and Abdel Raheem et al. of three injections combined with VED.^10^, ^12^ In a large multicentre study of 135 patients, a reduction of curvature of 19.1° (*p* < 0.001) was established using the modified scheme in 94.8% of patients.^24^ El‐Khatib et al. treated 21 men with stable PD with CCH. The overall mean improvement in curvature was 19° (*p* = 0.0079). Sixty‐four per cent reported subjective improvement of deformity.^25^ Ziegelmann et al. also found a mean improvement in curvature of 19° following treatment with CCH.^26^ Anaissie et al. retrospectively reviewed satisfaction of patient and partner after CCH treatment with a questionnaire. They included 24 patients, 16 patients reported overall satisfaction of treatment and 17 partners were satisfied with the treatment. There were no differences between satisfied and not satisfied patients.^27^

The PROM results show that reduction of the curvature leads to the possibility of sexual intercourse in 74.5% of patients after treatment. It also shows a treatment satisfaction of 54.9%. Reduction of the curvature causes 54% of the sexual partners to be more content as well. This demonstrates that despite a limited residue of curvature, the ability to perform intercourse again is paramount to treatment satisfaction. These findings add to the literature, focussing on satisfaction of the couple and having the ability to have sex again after treatment. The PROM can be developed to a standardized form to regular follow‐up on satisfaction of patient and partner after treatment, possibly also after surgical treatment.

A strength of this paper is that all injections were administered by the same urologist, and this paper reports on a large group of patients (*n* = 51) who received the modified scheme of three injections combined with the VED, according to the study of Abdel Raheem et al.^12^ Transition to the modified scheme of three injections was made because the reported results were similar to the original scheme.^12^ Of the 12 patients treated with the original scheme, eight (67%) completed the treatment of eight injections. Of the 51 patients treated with the modified scheme, 46 (90%) completed the treatment.

Another strength of our study is that the curvature of the penis was measured independently by three researchers which increases reliability. This is underlined by the intraclass correlation coefficient of curvature evaluation, showing no significant difference between researchers.

The present paper exhibits some limitations that have to be taken into consideration. The PDQ‐NL validation is pending, and the results and publication are expected to be published. The PDQ questionnaire can only be completed if vaginal intercourse is possible. As such, only the patients who could perform vaginal intercourse prior to and following treatment could be included in the analysis. This is a shortcoming of the PDQ and a cause of selection bias.

Furthermore, the applied PROM questions have not been validated, which is another limitation. Not all the patients answered all the PROM questions, which resulted in missing data, potentially another source of selection bias. Some patients did not have a sexual partner and so could not complete all questions.

Because patients photographed their own erect penis, the quality of the imaging was inconsistent. Not all patients provided pictures, despite several requests. These factors negatively affect the accuracy of the measured curvature.

Several patients experienced worsening of the curvature following treatment. This can be caused by calcification of the plaque prior to treatment.^28^ In the IMPRESS study, patients with calcified plaques on ultrasound were excluded from the study. Despite the recruitment of patients with (potentially) calcified plaques, the same reduction in curvature was observed.^13^

Another limitation concerns the applied VED. Use of VED twice daily showed 5° to 25° improvement of curvature in 66% after 3 months by Abdel Raheem et al. MacDonald et al. found 23° improvement of curvature after use of VED in 44% of patients.^29^, ^30^

According to the modified scheme, patients were asked to use the VED twice daily as in the study of Abdel Raheem et al.^12^, ^29^ The patients had to buy the VED themselves, which means the quality and brand was not uniform. Due to difference in quality, the use and results may have varied between patients. Furthermore, not all patients used the VED as recommended. This may have had a negative effect on the improvement of the curvature.

Despite injections being painful, the majority of the patients (96.1%) would recommend treatment with CCH. A possible explanation is that mainly patients with an aversion to surgical solutions sought CCH treatment. Even if the improvement of the curvature was minimal, sexual intercourse rates improved, possibly due to reduction in plaque size and hardness, causing the erect penis to bend more easily into the vagina.

In this study, the overall pain score was 4.3 justifying extra pain medication. Some patients received a lidocaine 2% penile block on request.

In the group treated with the modified scheme, five patients were motivated to receive an extra cycle of three injections, and they showed no further reduction of the curvature after the second cycle of three injections. Capece et al. reported that 5/17 patients receiving the second cycle of three injections had a further improvement of the curvature.^16^ Following treatment with CCH, only two (3%) patients underwent cavernoplication because of disappointing results.

Despite the mentioned limitations, this report on the outpatient CCH treatment in patients with PD demonstrates that CCH treatment effectively reduces curvature, increases the ability to perform penetrative sexual intercourse and is a safe treatment option.

Unfortunately, CCH is not available in Europe and Canada anymore, so this treatment option is not offered anymore in daily clinical practice.^31^, ^32^ This is the reason no further patients could be included in this analysis. Despite its unavailability, patients with PD frequently request a conservative treatment options such as CCH. This underlines the large demand amongst PD patients for treatment with CCH.

## 5 CONCLUSIONS

Intralesional treatment with CCH in men with PD leads to a mean curvature improvement of 20.6°. In following treatment, 74.5% of men were able to have sexual intercourse compared with 41.2% prior to treatment; 54.9% of the couples were satisfied with their sex life following treatment. No major complications occurred in the patients treated with CCH.

## DISCLOSURE OF INTEREST

The authors have no conflict of interest to report.

## AUTHOR CONTRIBUTIONS

J.P. Geelhoed is the primary researcher, PhD student. O. Wegelin, PhD, MD, has contributed by giving feedback on the content and English writing. E. Tromp, epidemiologist, has reviewed and contributed in the statistical method and results. Dr. B.‐J. de Boer, MD, has contributed in the measurement of photographs with the curvatures. Prof. Dr. I. J. de Jong, MD, is the promoter and coreader of the manuscript. Dr. J. J. H. Beck, MD, is the urologist performing the injections and measurement of curvatures, reading and giving feedback on the content and the copromoter.
